# Green Synthesis of Zn(OH)_2_/ZnO-Based Bionanocomposite using Pomegranate Peels and Its Application in the Degradation of Bacterial Biofilm

**DOI:** 10.3390/nano12193458

**Published:** 2022-10-03

**Authors:** Shafiul Haque, Hani Faidah, Sami S. Ashgar, Turki S. Abujamel, Jawahir A. Mokhtar, Mohammed Saad Almuhayawi, Steve Harakeh, Rajeev Singh, Neha Srivastava, Vijai Kumar Gupta

**Affiliations:** 1 Research and Scientific Studies Unit, College of Nursing and Allied Health Sciences, Jazan University, Jazan 45142, Saudi Arabia; 2 Department of Microbiology, Faculty of Medicine, Umm Al-Qura University, Makkah 24382, Saudi Arabia; 3 Vaccines and Immunotherapy Unit, King Fahd Medical Research Center, King Abdulaziz University, Jeddah 21589, Saudi Arabia; 4 Department of Medical Laboratory Technology, Faculty of Applied Medical Sciences, King Abdulaziz University, Jeddah 21589, Saudi Arabia; 5 Department of Medical Microbiology and Parasitology, Faculty of Medicine, King Abdulaziz University Hospital, Jeddah 21589, Saudi Arabia; 6 Vaccines and Immunotherapy Unit, King Fahad Medical Research Center, King Abdulaziz University, Jeddah 21589, Saudi Arabia; 7 Department of Medical Microbiology and Parasitology, Faculty of Medicine, King Abdulaziz University, Jeddah 21589, Saudi Arabia; 8 King Fahd Medical Research Center, Yousef Abdullatif Jameel Chair of Prophetic Medicine Application, Faculty of Medicine, King Abdulaziz University, Jeddah 21589, Saudi Arabia; 9 Department of Environmental Studies, Satyawati College, University of Delhi, Delhi 110052, India; 10 Department of Chemical Engineering and Technology, Indian Institute of Technology (BHU), Varanasi 221005, India; 11 Biorefining and Advanced Materials Research Center, SRUC, Kings Buildings, West Mains Road, Edinburgh EH9 3JG, UK; 12 Center for Safe and Improved Food, SRUC, Kings Buildings, West Mains Road, Edinburgh EH9 3JG, UK

**Keywords:** nanomaterials, food waste, green synthesis, bacterial biofilm degradation, zin oxide-based nanostructure

## Abstract

The ability and potency of bacterial species to form biofilms, which show antibiotic resistance thereby avoiding antibiotic surfaces, is a major cause of prolonged infections. Various advanced approaches have been employed to prevent or damage bacterial biofilms, formed by a variety of bacterial strains, to help prevent the associated infectious disease. In this context, zinc-based nanostructures have been recognized as a potential antibiotic agent against a broad spectrum of bacterial communities. As a result, a sustainable and green synthesis method was adapted in the present study to synthesize a Zn(OH)_2_/ZnO-based bionanocomposite, in which aqueous extracts of waste pomegranate peels (*Punica granatum)* were employed as a natural bioreducing agent to prepare the bionanocomposite at room temperature. Furthermore, FT-IR, XRD, DLS, UV-Visible, PL spectroscopy, FE-SEM, and TEM were used to characterize the green route synthesized a Zn(OH)_2_/ZnO bionanocomposite. The average crystallite size was determined using the Scherrer relation to be 38 nm, and the DLS results indicated that the Zn(OH)_2_/ZnO bionanocomposite had a hydrodynamic size of 170 nm. On the other hand, optical properties investigated through UV-Vis and PL spectroscopy explored the energy bandgap between 2.80 and 4.46 eV, corresponding to the three absorption edges, and it covered the blue spectrum when the sample was excited at 370 nm. Furthermore, the impact of this green route synthesized a Zn(OH)_2_/ZnO bionanocomposite on the biofilm degradation efficiency of the pathogenic bacterial strain *Bacillus subtilis* PF_1 using the Congored method was investigated. The Congored assay clearly explored the biofilm degradation efficiency in the presence of a 50 mg/mL and 75 mg/mL concentration of the Zn(OH)_2_/ZnO bionanocomposite against the bacterial strain *Bacillus subtilis* PF_1 grown for 24 h. This study can be further applied to the preparation of bionanocomposites following a low-cost green synthesis approach, and thus prepared nanostructures can be exploited as advanced antimicrobial agents, which could be of great interest to prevent various infectious diseases.

## 1. Introduction

Because of the distinctive physicochemical characteristics of nanomaterials, nanotechnology has substantial applications in many different domains of science and technology [1,2]. Fabricated nanomaterials could interact with biological molecules at the nanoscale and perform significant improvement in their efficiency [3]. Among various engineered nanomaterials, zinc-based nanomaterials (e.g., ZnO) are frequently used due to their numerous interesting properties such as optical, electrical, catalytic, and antimicrobial activities, along with their biocompatible non-toxic and eco-friendly nature [4,5,6]. Moreover, the biological activities, e.g., antimicrobial activity, of ZnO NPs are due to their unique size and morphology-dependent properties, and a number of studies have reported the highly promising antimicrobial activity of ZnO NPs against several microorganisms. This may help to prevent biofilm formation, and thereby act as an antimicrobial agent, which can be of great importance for a variety of biomedical applications [7]. Furthermore, a number of ZnO-based nanostructures have been extensively studied for their antibacterial efficacy against different types of microorganisms, including bacteria [8,9]. Additionally, the size, shape and morphology of ZnO nanostructures significantly relies on the synthesis methods employed therein [5]. Synthesis of ZnO-based nanostructures can be performed by following diverse physical, chemical, or biological methods. However, chemical synthesis routes to synthesize ZnO-based nanostructures are found to be toxic, cost-intensive, and complex. Additionally, chemical synthesis routes involve the release of hazardous gases that cause environmental pollution, and require expensive chemical reagents and equipment [10]. 

On the other hand, biological synthesis routes of nanomaterials offer several advantages, such as easy preparation methods, non-toxic synthesis route, clean, cost-effective approach, and involve fewer complex protocols to prepare a wide range of nanostructures [11]. A number of fruit-based extracts have been reported for agreen route synthesis of nanomaterials. Additionally, various fruit-based metabolites have been reported to perform diverse functions such as reducing, stabilizing, and capping agents during the synthesis of nanomaterials [12]. Different types of fruit wastes have been widely exploited to prepare ZnO-based nanostructures. In a study, the synthesize of ZnO NPs has been reported by using aqueous extracts of *Myristica fragrans*, and the prepared ZnO NPs exhibited diverse biomedical properties, e.g., antibacterial, antidiabetic, antioxidant, antiparasitic, and larvicidal [13]. Okpara et al. [14] reported the synthesis of ZnO NPs using different citrus peel wastes, including grape (*Citrus x paradise*), lemon *(Citrus limon)* and orange (*Citrus sinensis*), which showed improved electro-catalytic properties [14]. Khan et al. [15] reported using *Passifora foetida* fruit peel extract to synthesize ZnO NPs following a controlled ultrasound cavitation approach and investigated the photocatalytic activity to degrade dyes, e.g., methylene blue and rhodamine [15]. Similarly, orange and banana peel waste were employed as bioreducing phytochemicals to synthesized ZnO NPs which showed size, shape and morphology-dependent antibacterial activities [16]. Ravichandran et al. [17] utilized *Durio zibethinus* fruit waste (rind) to prepare ZnO NPs and investigated their antibacterial, antioxidant, cytotoxicity, and photocatalytic efficiency properties [17]. *Garcinia mangostana* fruit pericarp extract was employed as a simultaneous reducing and capping agent to prepare ZnO NPs, and the photocatalytic properties to degrade malachite green were probed [18]. In a recent study, Abdelmigid et al. [19] utilized pomegranate (*Punica granatum* L.) peel and coffee ground (*Coffea arabica*) extract to prepare ZnO NPs [19]. These authors reported antibacterial efficiency against different bacterial strains and also reported that green route synthesized ZnO NPs exhibited a lower cytotoxicity on Vero cells compared to ZnO NPs synthesized by a chemical route. Thus, it is noted that fruit wastes can be of great importance whenpreparing a wide range of nanomaterials following a green synthesis approach, and thus prepared nanostructures can be exploited for numerous technological applications.

Among various fruits, pomegranate (*Punica granatum* L.) is one of the most valuable fruit crops that has been recognized based on its nutritional properties, and rich in several bioactive compounds like polyphenolics. Since it is found to be rich in a number of phytochemicals, this fruit crop has had broad industrial applications in food- and therapeutic-related industries [20]. Additionally, pomegranate peel (Pp) is considered one of the agro-wastes with wide availability, covering around 40–50% of the total weight of the fruit [21]. Further, Pp contains potential reactive oxygen species (ROS) owing to being enriched with bioactive compounds, namely polyphenols with gallic acid and tannins, which amount toaround ~25–28% of the total portion [22]. Moreover, polyphenolsare one of the major components of pomegranates which exhibit an antimicrobial biofilm degradation efficiency against many bacterial diseases [23].

Furthermore, the presence of layered types of nanostructures, forming the nanocomposite crystalline structure exhibit unique physicochemical properties that can improve the catalytic efficiency of a nanomaterial. These layered double hydroxide nanostructures can also be used as antibacterial agents [24]. Wu et al. [25] reported preparation of ZnO/Zn(OH)_2_ by hydrothermal method, and exhibited superior photocatalytic properties for H_2_ generation compared to bare ZnO nanorods [25]. These researchers discovered that in such composite structures, layered Zn(OH)_2_ acted as a structural component, helping to improve surface area, while ZnO only acted as a functional component. Faheem et al. recently reported a chemical method for producing ZnO/Zn(OH)_2_ NPs via surface protonation of pre-synthesized ZnO NPs. Furthermore, when compared to bare ZnO NPs, ZnO/Zn(OH)_2_ demonstrated a 3.5-fold improvement in photocatalytic efficiency for the degradation and removal of sunset yellow dye [26]. Furthermore, such ZnO/Zn(OH)_2_ nanostructures can be used as a hard template to prepare a wide range of nanomaterials with diverse physicochemical properties, such as high specific surface area, crystallinity, and mesoporosity, thus exhibiting improved catalytic efficiency [27]. Nonetheless, a literature review revealed that only a few studies on the preparation of Zn(OH)_2_/ZnO have been reported, and no study on the application of Zn(OH)_2_/ZnO-based nanostructures on bacterial biofilm degradation has been published.

Given this, the current study aims to accomplish the following objectives: (i) green synthesis of a photoactive Zn(OH)_2_/ZnO bionanocomposite from pomegranate peels; (ii) physicochemical characterizations of a Zn(OH)_2_/ZnO bionanocomposite; and (iii) biofilm degradation efficiency of a Zn(OH)_2_/ZnO bionanocomposite formed by *Bacillussubtilis* PF_1. The bacteria *Bacillus subtilis*PF_1, which is a very common surface pathogen, is responsible for many healthcare issues, a potential biofilm producer, and very resistant to a broad range of antibiotics.

## 2. Materials and Methods

### 2.1. Materials & Consumables

The analytical grade chemicals and reagents were procured from the local market. As the precursor of zinc, 99% pure zinc sulphate heptahydrate (ZnSO_4_·7H_2_O) as a zinc metal salt was purchased from the Merck, Sigma Aldrich, Mumbai, India.

### 2.2. Sampling of Fruit Waste Materials and Extract Preparation

Waste peels of pomegranate (Pp) fruits were first collected and then washed several times using tap water. Subsequently, washing was performed using the deionized water, and then peels were wiped with cotton cloths. Further, Pp samples were sun dried followed by oven drying at 60 °C until completely dry. The Pp samples were then crushed and ground to a fine powder by a grinder, and then a moisture analysis was conducted using a digital moisture analyzer. Following that, 10 g of completely dried sample powder was mixed in 90 mL of double deionized (DD) H_2_O and stirred continuously at 100 °C until the volume of the mixture was reduced by half. Subsequently, the obtained liquid mixture was allowed to naturally cool and was then filtered through Wattman filter paper. The filtered liquid was directly used as a reducing agent to synthesize bionanocomposite.

### 2.3. Green Synthesis of Zn(OH)_2_/ZnO Bionanocomposite 

**Figure 1:**
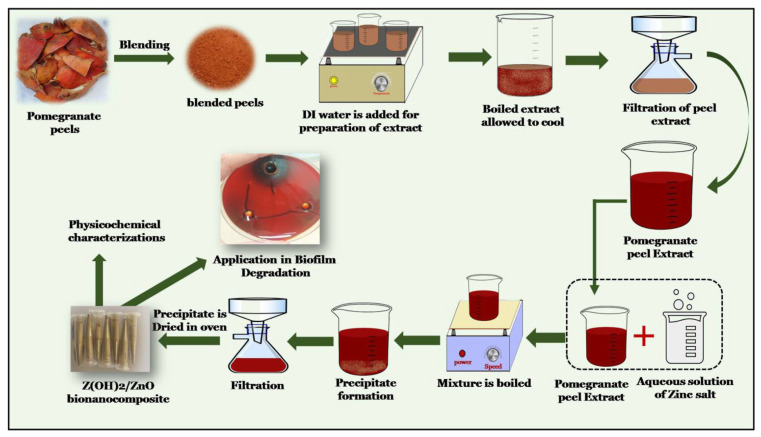
Schematic diagram for the preparation of the Zn(OH)_2_/ZnO bionanocomposite.

For the synthesis process, 50 mL of Pp extract was taken at room temperature and, simultaneously 25 mL of 1.0 M prepared solution of ZnSO_4_·7H_2_O was slowly added. Furthermore, the mixture was kept at 60 °C on a magnetic stirrer for continuous stirring for 1h. After this, the reaction mixture was cooled down and monitored for color changes and precipitation formation. This was followed by precipitate filtration, which was allowed to completely dry in an oven at 60 °C. The completely oven dried material was then crushed to a fine powder and then used for the characterization studies. Figure 1 shows the schematic diagram for the overall preparation method of bionanocomposite. 

### 2.4. Characterizations of the Zn(OH)_2_/ZnO Bionanocomposite

The prepared sample was characterized using various techniques such as Fourier transform infrared (FT-IR) spectroscopy [Nicolet iS5, THERMO Electron Scientific Instruments LLC, Waltham, MA, USA], powder X-ray diffraction pattern (XRD) [Ultima IV, X-ray diffractometer, Rigaku Corporation, Tokyo, Japan], dynamic light scattering (DLS) [Zetasizer Ultra (ZSU 5700) Malvern Panalytical (Malvern, UK)], ultraviolet-visible (UV-Vis) spectroscopy [Model no LMPS-UV1900, Make LABMAN, North Yorkshire, UK], photoluminescence (PL) spectroscopy [Horiba Fluoromax 4CP plus spectrofluorometer, Kyoto, Japan], field emission scanning electron microscopy (FE-SEM) [Nova Nano SEM 450, FEI Company of USA (S.E.A.) PTE, LTD., Jalan Kilang Timor, Singapore], and transmission electron microscope (TEM) [Tecnai G2 20 TWIN, FEI Company of USA (S.E.A.) PTE, LTD., Jalan Kilang Timor, Singapore] to analyze its physicochemical properties.

### 2.5. Bacterial Strain Cultivation Condition and Anti-Biofilm Study

The commonly known pathogenic bacteria *Bacillus subtilis*PF_1 was screened by a serial dilution method, and then identified by the molecular characterization technique named 16S. The accession number for this strain of bacteria was MG593077, and it was used as a model microbial system in this study. More detail about the molecular characterization study of *Bacillus subtilis*PF_1 has been provided in our earlier study [28]. This bacterial culture was grown and developed in potato-dextrose broth and agar medium at 37 °C. Further, preservation of this bacterium was carried out in a 3.0% glycerol stock solution under sterilized conditions.

Using the methods described by Freeman et al. [29], the anti-biofilm efficiency of the synthesized Zn(OH)_2_/ZnO bionanocomposite was investigated. Herein, a slight modification in the combination of nutrient agar (NA) plate and 1.0% Congo red was used to analyze the formation of a biofilm. The NA plate was inoculated with the bacteria and incubated at 37 °C for 24 h. Following this, plates were stained with 1.0% Congo red dye for 20 min and then de-stained with 1.0% NaCl solution. Moreover, the biofilm degradation efficiency was analyzed by the investigation of black colonies with a dry crystalline consistency. Additionally, bacterial colonies with weak slime production generally remained pink or sometimes darkened.

### 2.6. Statistical Analysis

Experiments were performed in triplicate to investigate the anti-biofilm degradation efficiency of the Zn(OH)_2_/ZnO bionanocomposite, and standard deviations were calculated using the Microsoft Excel software 2007.

## 3. Results &Discussion

### 3.1. Characterizations

**Figure 2:**
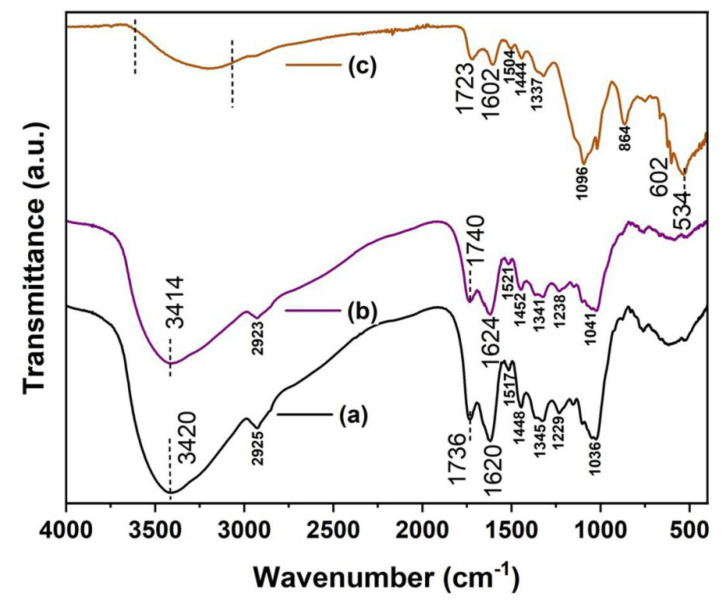
FT-IR spectra of (**a**) pomegranate peels powder (**b**) aqueous extract from pomegranate peels powder, and (**c**) Zn(OH)_2_/ZnO bionanocomposite.

FT-IR spectra have been recorded and analyzed to identify various functional groups which correspond to the presence of different bioactive components. The FT-IR spectra of *Punica granatum* peel powder and aqueous extract are shown in Figure 2a,b. The spectra showed a number of peaks and suggesting the complex nature of both samples. Moreover, careful analysis showed that both the spectra show similar patterns except for minor variations in the peak positions. A strong peak was observed at ~3414 cm^−1^, a characteristic of alcohol/phenol-OH stretching vibrations, carboxylic acid-OH stretching, and N-H stretching of amides. However, this may also be attributed to the O-H stretching corresponding to the presence of water molecules. The CH_2_/CH_3_ stretching is responsible for the observed low intensity peak at 2923 cm^−1^. On the other hand, the presence of an intense peak observed at 1740 cm^−1^ correlates with C=O stretching or C=N bending corresponding to either the carboxyl or amide functional groups, respectively. Additionally, the presence of a strong peak observed at 1624 cm^−1^ is attributed to N-H stretching, caused by the presence of primary amines, whereas low intensity peaks recorded at ~1521 cm^−1^, 1452 cm^−1^, 1341 cm^−1^ and 1238 cm^−1^ can be associated with the –NH in secondary amines, aromatic -CH stretching vibrations, and C-C-N amines. A strong peak observed at 1041 cm^−1^ is attributed to the stretching vibration of (NH)–C-O. These observations are in good agreement with earlier work [30]. It is worthy to mention that these functional groups can effectively act as reducing agents to reduce metal salts. Furthermore, they may act as capping agents during the growth phase of nanostructures. The FT-IR spectrum of the prepared bionanocomposite sample (Figure 2c) exhibited a number of peaks which are very similar to the FT-IR spectrum as shown in Figure 2a,b. Nevertheless, the intensity of these peaks can also be seen to be reduced. Peaks that appeared at 602 cm^−1^ and 534 cm^−1^ can be attributed to the Zn-OH and Zn-O bonds in the crystalline structure of the synthesized bionanocomposite [31,32,33].

**Figure 3:**
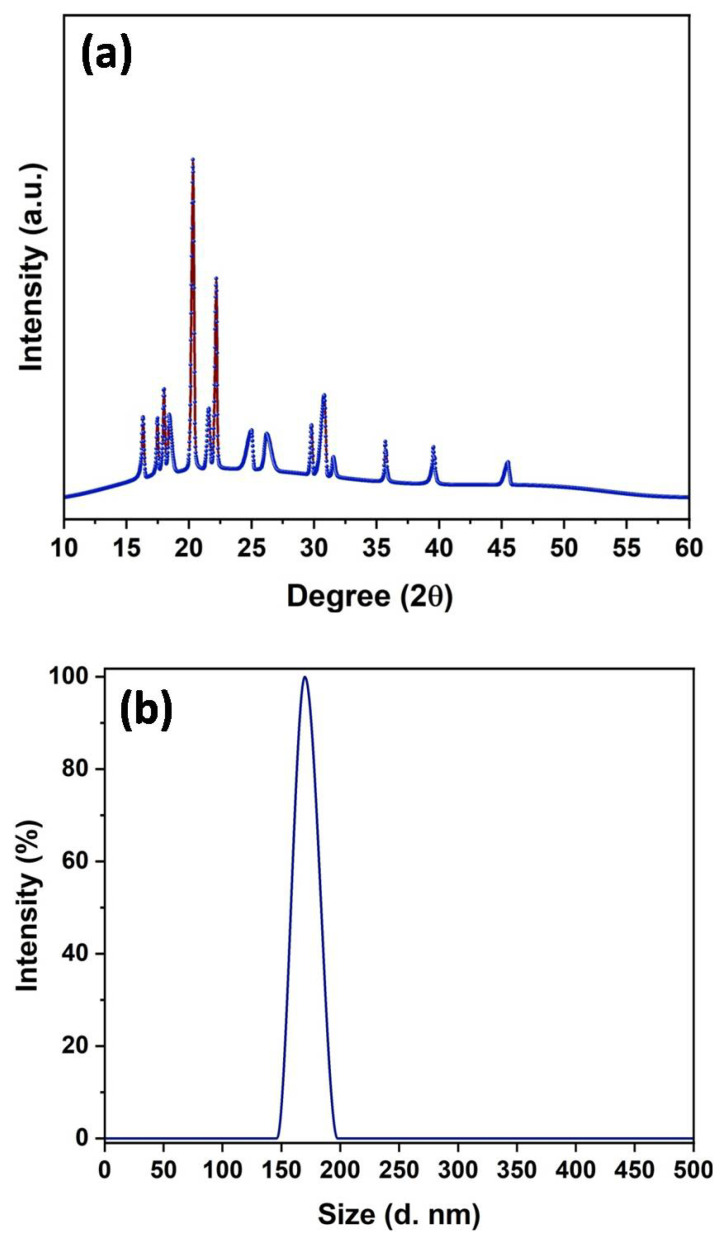
(**a**) XRD pattern, and (**b**) DLS spectra of synthesized sample.

The XRD pattern was recorded to analyze the crystalline structure and phase formation in the synthesized sample, and the spectra are shown in Figure 3a. It was noted that the XRD spectrum showed a number of peaks, wherein some of the peaks were found to be very intense, along with several peaks having a relatively low intensity. The high intensity of diffraction peaks suggested a crystalline nature of the prepared sample. These diffraction peaks matched well with the corresponding ε/γ/β crystalline phases of Zn(OH)_2_, while a few diffractions peaks corresponding to the hexagonal wurtzite ZnO crystalline phase were also recorded. Thus, XRD analysis revealed that the sample primarily composed of Zn(OH)_2_ with a small fraction of ZnO, implying the formation of a Zn(OH)_2_/ZnO bionanocomposite [34,35,36]. Furthermore, average crystallite size was calculated by using the Scherrer formula and found to be 38 nm. The DLS technique was used to determine the hydrodynamic size of the Zn(OH)_2_/ZnO bionanocomposite, and the average size was determined to be 170 nm (Figure 3b).

**Figure 4:**
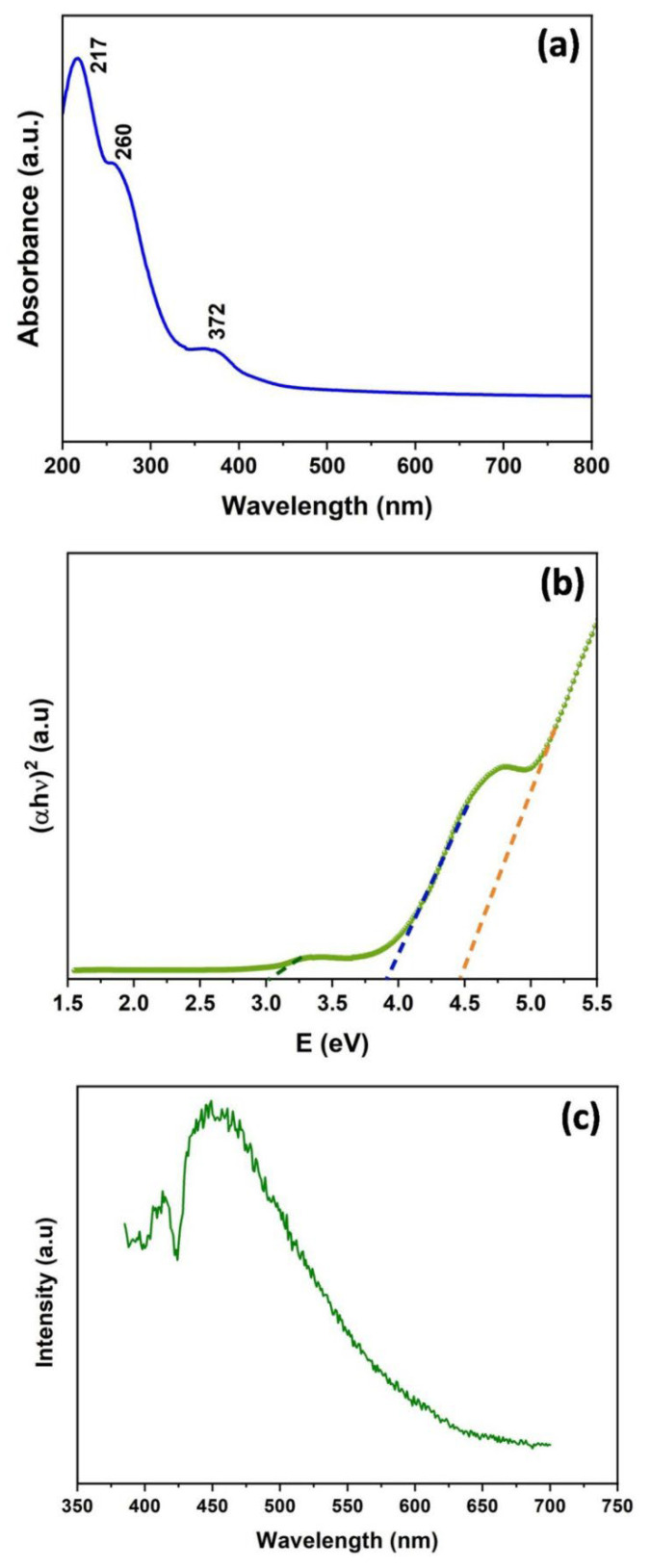
(**a**) UV-Vis absorption spectrum (**b**) Tauc plot (**c**) PL spectrum of the Zn(OH)_2_/ZnO bionanocomposite at the excitation wavelength 370 nm.

The optical properties were analyzed through UV-Vis spectroscopy, and the spectrum recorded between 200 and 800 nm is presented in Figure 4a. The UV-Vis spectrum exhibited three peaks with the highest absorption at 217 nm, 260 nm, and 272 nm. These peaks may be attributed to the three different optical band gaps corresponding to the different phases present in the Zn(OH)_2_/ZnO composite nanostructure [37,38]. In a study, Wang et al., also recorded two absorption peaks located at ~219 nm and 368 nm, and reported that these peaks were correlated to the band edge absorptions of Zn(OH)_2_ and ZnO phases, respectively [39]. Additionally, these authors reported that the calculated band gap (E_g_) corresponding to the Zn(OH)_2_ (5.65 eV) and ZnO (3.37 eV) phases were well supported by the theoretically calculated values. Thus, the UV-Vis spectrum suggested that both Zn(OH)_2_ and ZnO phases were present in the prepared bionanocomposite sample. It is worthy to mention that the additional peak observed at ~260 nm may be due to the quantum confinement in the form of ZnO nanoparticles compared to the bulk ZnO [40]. Nevertheless, this peak may also be associated with n–π* transition caused by the presence of biological molecules on the surface of the Zn(OH)_2_/ZnO bionanocomposite, and this phenomenon is supported by the FT-IR analysis as discussed above. Such observations have been made in the study reported by Rajendran et al. [41]. Herein, the optical band gap was calculated using the Tauc relationship, assuming a direct band gap between the valance and conduction bands. The Tauc plot is shown in Figure 4b. It was noted that band gaps corresponding to the three edges observed in the UV-Vis spectrum with values of 2.80 eV, 3.44 eV, and 4.46 eV were calculated from the Tauc plot. These observations were in good agreement with studies reported by Ekennia et al. [42] and Faheem et al. [26]. These authors reported 2.72–4.37eV band gap in the case of biogenic ZnO nanostructures synthesized using *Euphorbia sanguinea* as the reducing agent.

The emission behavior of the synthesized Zn(OH)_2_/ZnO bionanocomposite was investigated using a room temperature PL spectrum with an excitation wavelength of 370 nm. The PL spectrum, as shown in Figure 4c showed a broad visible emission band which extended over 423–500 nm. This spectrum nearly covered the blue emission region, attributed to the intrinsic defects which may arise due to the oxygen and/or zinc vacancies or the interstitial Zn defects [43]. This observation is in good agreement with the study reported by Jose et al. [44]

**Figure 5:**
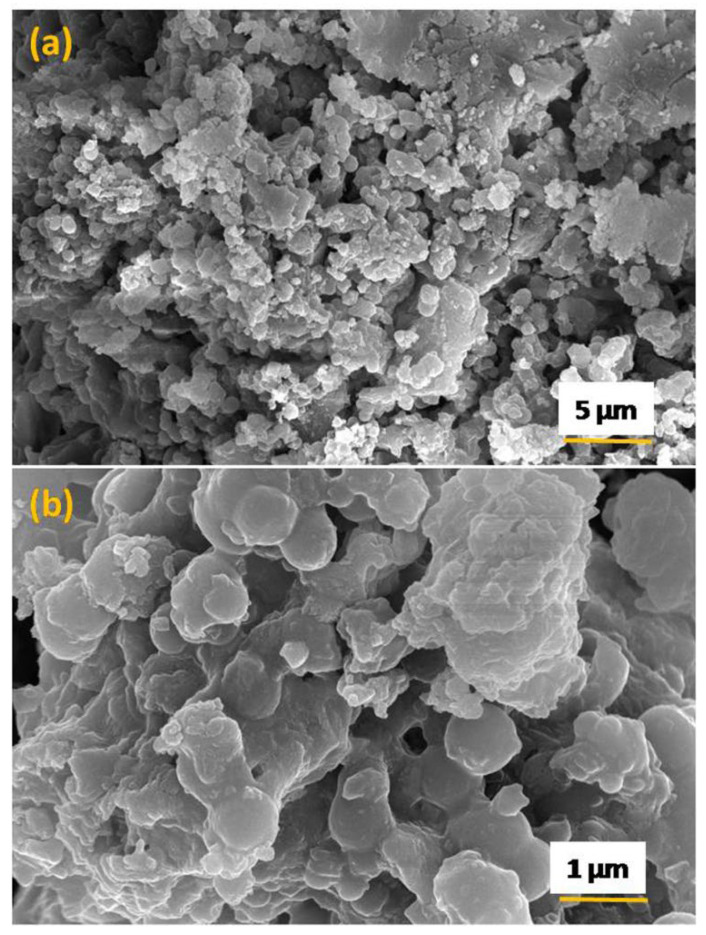
(**a**,**b**) FE-SEM micrographs of the Zn(OH)_2_/ZnO bionanocomposite at two different magnifications.

**Figure 6:**
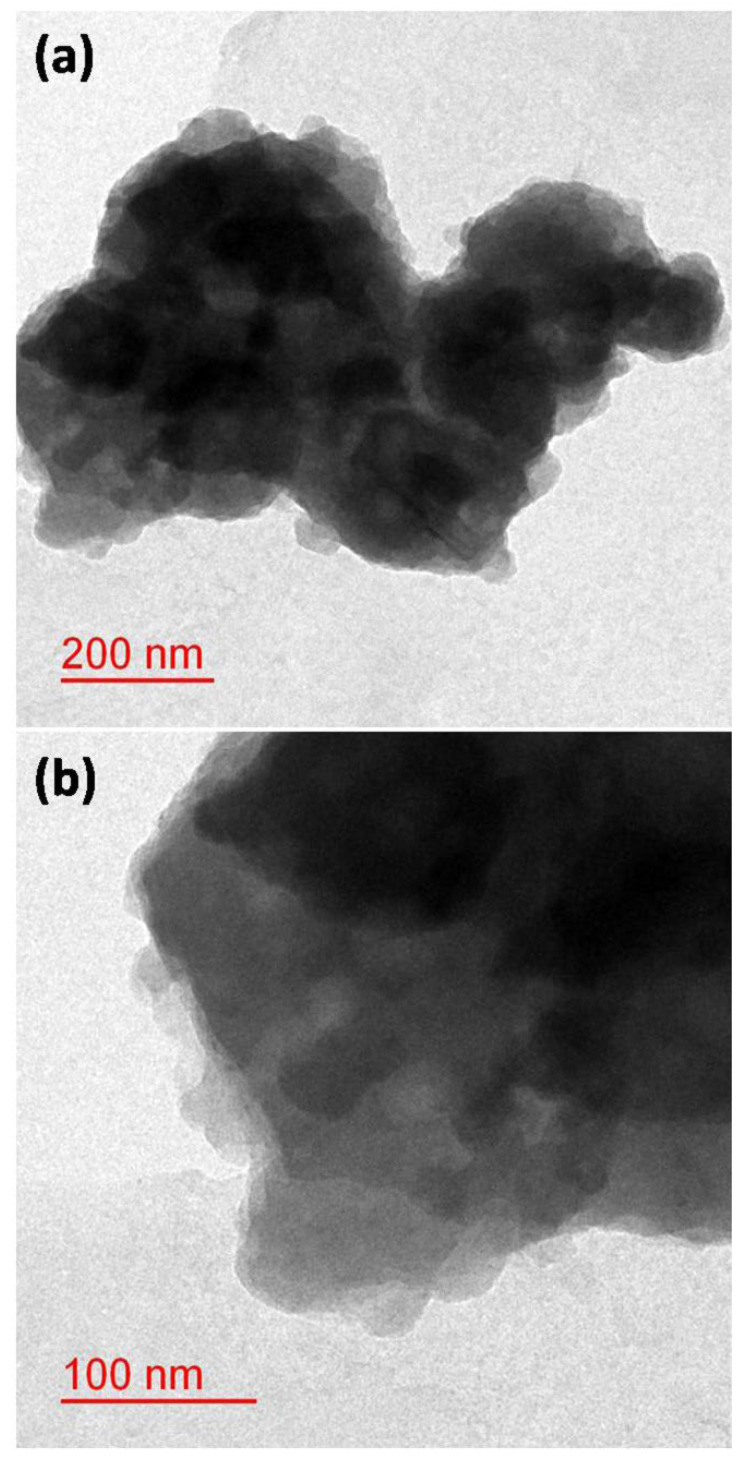
(**a**,**b**) TEM micrographs of Zn(OH)_2_/ZnO bionanocomposite taken at two different magnifications.

The surface morphology of the sample was probed through the FE-SEM technique, and micrographs are shown in Figure 5a,b. It was noticed that particles consisting of a sheet type structure which together form a spherical-type hierarchical structure. In addition, TEM micrographs, as shown in Figure 6a,b, further explored the spherical hierarchical structure. 

### 3.2. Biofilm Degradation

**Figure 7:**
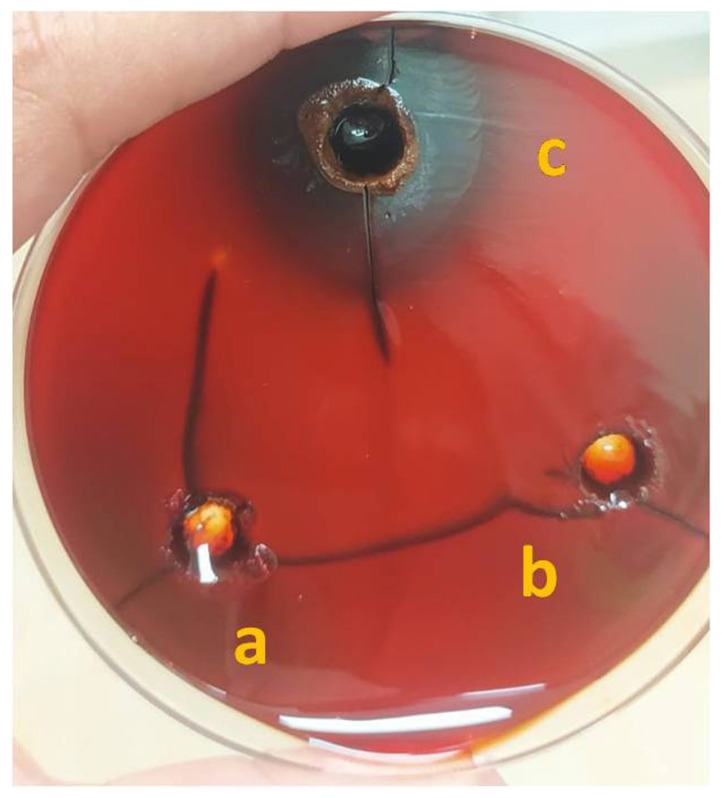
Photograph shows bio-film degradation efficiency of the Zn(OH)_2_/ZnO bionanocomposite using Congored method at a concentration of (**a**) 50 mg/mL (**b**) 75 mg/mLand (**c**) control.

Biofilms consist of an exopolysaccharide matrix which is synthesized by numerous bacterial microorganisms, and this polymeric matrix provides protection to bacterial colonies during the transition phase as well as under adverse environmental conditions. It has been reported that more than 60% of all infections are caused by the formations of biofilms synthesized by bacterial microorganisms [45]. Moreover, biofilm producing microorganisms have an inherent resistance ability towards antibiotics, disinfectants, and germicides; therefore, in depth research is needed to offer advanced therapeutic strategies [46]. There are few well-known anti-biofilm compounds that act against different microbial strains. Among various bacterial stains, *Bacillus subtilis*PF_1 is known to be a potential producer of bacterial biofilms [47]. Further, to break down the bacterial biofilm and impede its formation, antimicrobials agents must penetrate the polysaccharide matrix to enter to the bacterial cells. This function could be performed by using different types of nanostructures; however, this phenomenon significantly relies on the unique physicochemical properties of the nanomaterial [48]. The efficacy of the Zn(OH)_2_/ZnO bionanocomposite to inhibit the formation of bacterial biofilms was investigated in this study using the CRA method, and the results are shown in Figure 7. The presence of dark black stained colonies indicated bacterial biofilm formation, as seen in 24 h grown bacterial colonies in the absence of the synthesized Zn(OH)_2_/ZnO bionanocomposite. There was no formation of an exopolysaccharide matrix when the Zn(OH)_2_/ZnO bionanocomposite was used at concentrations of 50 mg/mL and 75 mg/L, indicating that the synthesized Zn(OH)_2_/ZnO bionanocomposite successfully blocked the formation of a biofilm. Divya et al. [49] also reported such observations, wherein these authors investigated the antibiofilm activity of chitosan nanoparticles against different medically recognized pathogens, e.g., *E. coli*, *K. pneumonia* and *P. aeruginosa* [49]. The present results are in accordance with the study by Ansari et al. [48]. These authors investigated the potential of Ag NPs in biofilm degradation against bacterial strains *E. Coli* and *K. pneumonia*. Similar results were reported in the study by Kalishwaralal et al. [50], wherein the potency of Ag NPs was evaluated against the biofilms formed by bacterial strains *P. aeruginosa* and *S. epidermidis* [50]. However, significant effort should made in the preparation of novel nanostructures using low-cost green synthesis methods, along with considering their size, shape, and morphology-dependent antibiofilm efficacy. This could offer high efficiency anti-biofilm agents, and be of great importance in the applications of nanomaterials-enabled therapeutic agents for a variety of biomedical applications [51].

## 4. Conclusions

This study described a simple green synthesis route for preparing a Zn(OH)_2_/ZnO bionanocomposite, in which pomegranate waste peels were used as a natural metal salt reducing agent. Furthermore, the synthesized Zn(OH)_2_/ZnO bionanocomposite was characterized using a variety of techniques to investigate its physicochemical properties. The combined investigation using FT-IR, FE-SEM, and TEM techniques revealed that the prepared Zn(OH)_2_/ZnO bionanocomposite had some hierarchical structure consisting of sheet type nanostructures, as well as the presence of bioactive compounds.Using the Congored method, the catalytic efficiency of the Zn(OH)_2_/ZnO bionanocomposite was investigated for the degradation of biofilms formed by the pathogenic bacteria *Bacillus subtilis*PF_1. When two different doses of the prepared bionanocomposite with concentrations of 50 mg/mL and 75 mg/mL, the bacterial biofilm degradation efficiency of the Zn(OH)_2_/ZnO bionanocomposite was clearly demonstrated by the Congo red method against the *Bacillus subtilis* PF_1 strain compared to the control. This study could be very helpful for making advanced bionanocomposite materials as novel antimicrobial agents, thus preventing a number of infectious diseases. 

## Data Availability

The data presented in this study are available on request from the corresponding author.
